# Polygenic risk meets early trauma: toward a neuropsychodevelopmental model of schizophrenia

**DOI:** 10.1007/s00702-026-03263-4

**Published:** 2026-08-21

**Authors:** Antonio Del Casale, Paolo Girardi, Jan Francesco Arena, Claudio Cantelmi, Martina Nicole Modesti, Liliana Bronzatti, Ludovica Zanini, Giorgio Veneziani, Emanuele Giraldi, Sofia Mazzani, Maurizio Simmaco, Carlo Lai, Grazia Spitoni, Giacomo Ciocca

**Affiliations:** 1 Department of Dynamic and Clinical Psychology and Health Studies, Faculty of Medicine and Psychology, Sapienza University of Rome, Rome, 00185 Italy; 2 Unit of Psychiatry, Policlinico Umberto I University Hospital, Rome, 00185 Italy; 3 Department of Human Neurosciences, Faculty of Medicine and Dentistry, Sapienza University of Rome, Rome, 00185 Italy; 4 Department of Neuroscience, Mental Health and Sensory Organs (NESMOS), Sapienza University of Rome, Rome, 00189 Italy; 5 Department of Wellbeing, Health and Environmental Sustainability, Sapienza University of Rome, Rome, 00185 Italy

**Keywords:** Schizophrenia, Genetic predisposition to disease, Psychological trauma, Psychosis, Prodromal symptoms, Affect, Epigenesis, genetic, Neurodevelopmental disorders

## Abstract

Advances in psychiatric genetics have substantially reshaped current models of schizophrenia-spectrum disorders, shifting the focus from single risk factors to polygenic liability and its interaction with environmental adversity. Polygenic risk scores (PRS) provide a quantitative index of genetic vulnerability; however, how this liability interfaces with early-life stress—particularly childhood trauma—across neurodevelopment remains insufficiently conceptualized. Accumulating evidence indicates that the interplay between polygenic risk and childhood trauma influences not only the likelihood of psychosis onset but also the emergence of specific symptom dimensions during clinical high-risk states and early stages of illness. Yet, existing theoretical frameworks tend to consider genetic predisposition and environmental exposure as parallel or additive processes, offering limited insight into their developmental convergence. In this Perspective, we examine current neurodevelopmental models of schizophrenia and integrate them within a unified framework, proposing that polygenic liability, environmental exposures (including early trauma), and psychological processes interact dynamically and bidirectionally across sensitive developmental windows, shaping distinct neuropsychodevelopmental trajectories. Integrating polygenic, environmental, and developmental domains within a unified neuropsychodevelopmental framework may help explain clinical heterogeneity, with important implications for early identification, risk stratification, and personalized preventive strategies.

## Introduction

Schizophrenia is among the most complex and burdensome mental disorders, characterized by a multifactorial etiology arising from the interplay of genetic, epigenetic, and environmental factors within a dense and dynamic risk network (Mujtaba et al. 2025). Twin and family studies have consistently demonstrated a substantial heritable component, with heritability estimates ranging between 80 and 85% (Hilker et al. 2018). Despite this strong genetic signal, early attempts to identify specific risk genes through candidate gene approaches or linkage studies produced largely inconsistent and poorly replicable findings (Farrell et al. 2015).

A major methodological shift occurred with the advent of genome-wide association studies (GWAS) (Tam et al. 2019), which enabled the systematic identification of an increasing number of common genetic variants associated with schizophrenia risk (Harrison 2015; Owen et al. 2023). Importantly, each of these variants confers only a minimal effect size, such that no single locus meaningfully accounts for disease liability. Rather, it is the cumulative contribution of thousands of variants that explains a substantial proportion of genetic risk (Owen et al. 2023). Within this framework, polygenic risk scores (PRS) have emerged as a quantitative tool to aggregate the effects of multiple genetic variants into an individual-level index of vulnerability to schizophrenia and related phenotypes (Kendler 2016; Choi et al. 2020).

Concurrently, growing attention has been devoted to environmental risk factors, particularly childhood traumatic experiences, which are now widely recognized as powerful determinants of psychopathological vulnerability across the lifespan (Albott et al. 2018; McLaughlin et al. 2020; Lemvigh et al. 2023). Early-life adversity has been conceptualized not merely as a risk factor, but as a differential susceptibility condition capable of shaping biological and psychological reactivity to later stressors, potentially amplifying both adaptive and maladaptive responses (Albott et al. 2018). The co-occurrence of genetic vulnerability and childhood trauma (Woolway et al. 2022; Alameda et al. 2024) raises critical questions regarding how these domains interact during development to bias trajectories toward psychosis.

Importantly, emerging evidence suggests that polygenic liability to schizophrenia may manifest early through dimensional and often non-psychotic features—such as negative symptoms, anxiety, and affective dysregulation—particularly during childhood, adolescence, and clinical high-risk states, rather than through overt positive psychotic symptoms (Myin-Germeys and van Os 2007; Isvoranu et al. 2017).

Within this perspective, psychosis is increasingly conceptualized as the potential outcome of partially distinct developmental pathways, including affective-reactive trajectories in which emotional dysregulation and stress sensitivity play a central role in the emergence of psychotic phenomena.

In this Perspective, we examine current models addressing genetic, environmental, and developmental contributions to schizophrenia, focusing on how polygenic risk and early traumatic experiences may converge across neurodevelopment. We propose a Neuropsychodevelopmental Model that integrates these perspectives, conceptualizing psychosis risk as the result of dynamic interactions among polygenic liability, early trauma, and developmental timing. Accordingly, the models reviewed below are presented as complementary components that converge toward this integrative framework.

## Discussion

### Current perspectives on schizophrenia development: toward an integrated developmental account

Schizophrenia can be conceptualized as the outcome of multiple liability streams—polygenic, environmental, and developmental—that do not simply accumulate but may interact across time to generate heterogeneous trajectories. In this context, the key challenge is not whether genetics or childhood trauma matters, but how polygenic liability and early adversity become biologically and psychologically embedded during development, and why their convergence appears to bias individuals toward distinct phenotypic expressions and symptom dimensions. The models reviewed below are therefore presented as complementary lenses through which to examine a shared question: how risk is organized across neurodevelopment to shape different pathways to psychosis. Rather than an exhaustive catalogue, they serve as conceptual building blocks that converge in the Neuropsychodevelopmental Model proposed at the end of this section.

### Polygenic and environmental risk in clinical high-risk (CHR) states

The PRS is calculated from a training sample (including cases and controls), in which the genetic variants associated with the condition of interest are identified and their effect sizes estimated. In the target sample, each individual is assigned an overall score based on the number of risk alleles present and the weights derived from the training sample (Kendler 2016; Choi et al. 2020). The schizophrenia PRS thus reflects an aggregate index of genetic vulnerability. While it does not allow for precise prediction of who will develop the disorder, it enables the exploration of how genetic predisposition influences intermediate phenotypes and psychopathological traits in the general population or in clinical high-risk states (Jones et al. 2016; Kendler 2016; Mistry et al. 2018).

Importantly, PRS associations in youth and CHR samples suggest that polygenic liability may manifest in dimensional and often non-psychotic phenotypes before frank onset. For example, a recent study found that, in an adolescent population, a high PRS for schizophrenia was associated with increased negative symptoms and anxiety disorders, but not with positive psychotic symptoms or depressive disorders (Jones et al. 2016). Individuals in the top 2.5% of PRS had a 45% increased risk of developing negative symptoms or at least one anxiety disorder during adolescence (Jones et al. 2016). Consistent with this dimensional perspective, the schizophrenia PRS and the environmental risk score (poly-environmental risk score), taken together, did not predict psychotic experiences in adolescence, either individually or in combination (Navarro et al. 2021).

These findings underscore two implications that align with the introduction’s emphasis on heterogeneity: first, polygenic risk may “surface” early through negative/disorganized or anxiety-related features rather than through positive psychotic experiences; second, environmental risk may not combine with PRS in a simple additive manner during adolescence. In CHR states, this matters because “riskiness” does not appear to be exclusively linked to transient psychotic experiences, but rather to the combination of such experiences with other non-psychotic mental disorders, such as anxiety, depression, or substance use (Hasmi et al. 2021). These observations support a dimensional model of psychopathology, in which genetic vulnerability to schizophrenia tends to manifest preferentially through negative and disorganized dimensions, while other psychopathological dimensions—particularly those related to affective symptoms—may be more closely associated with distinct genetic profiles, more akin to bipolar disorder or major depression (Hasmi et al. 2021).

### The role of childhood trauma and its interaction with polygenic risk for schizophrenia

Within the same dimensional and developmental lens, childhood traumatic experiences, such as physical abuse, emotional abuse, or neglect, represent a major risk factor for a wide range of mental disorders, including affective and psychotic conditions (Ciocca et al. 2021; Alameda et al. 2024). Early trauma exposure has been shown to triple the risk of developing psychiatric conditions over the lifespan and appears to influence both symptom severity and overall functioning in individuals with psychosis (Alameda et al. 2024). However, the relationship between childhood trauma and genetic vulnerability remains complex. Some studies have suggested a synergistic interaction, whereby trauma amplifies the phenotypic expression of genetic risk, while others have reported compensatory effects or even reversals in association patterns (Woolway et al. 2022; Alameda et al. 2024).

Only recently have we begun to understand the interactions between PRS, childhood trauma, and the onset of psychosis. A recent study by Alameda and colleagues, conducted on a sample of 583 patients with first-episode psychosis (FEP), examined PRSs for schizophrenia, major depressive disorder, and bipolar disorder in relation to childhood traumatic experiences and symptom dimensions at onset (Alameda et al. 2024). The PRS for major depressive disorder was negatively associated with both positive and negative symptom dimensions, with a significant interaction with childhood trauma for the positive dimension. In the presence of trauma, the association between PRS and positive symptoms was reversed, suggesting that traumatic experience may alter the expression of genetic risk. Similar results were observed for the bipolar disorder PRS, which also interacted with childhood trauma in predicting positive symptoms. In contrast, the schizophrenia PRS showed significant associations with symptom dimensions—though only a trend with the positive dimension—and exhibited a negative interaction with childhood trauma, opposite to the direction observed in the other two conditions (Alameda et al. 2024).

Taken together, these findings argue against a unitary “PRS × trauma” effect and instead support the introduction’s premise that distinct genetic liabilities may converge with trauma in qualitatively different ways. The observed interaction between genetic vulnerability to mood disorders and childhood trauma in shaping positive symptomatology at the onset of psychosis recalls the concept of the “affective pathway of psychosis” (Myin-Germeys and van Os 2007). According to this model, psychosis may emerge as the outcome of intense affective responses to stressful—even everyday—events in individuals genetically predisposed to emotional instability and heightened stress reactivity (Myin-Germeys and van Os 2007). This affective pathway differs from the well-established cognitive model of negative symptoms, which is characterized by neurocognitive impairment, affective flattening, dysfunctional beliefs, and negative symptoms (Saperia et al. 2025). In the more affectively driven trajectory, psychosis does not represent a monolithic entity but rather the result of distinct pathogenetic pathways converging on similar clinical outcomes. These data suggest that psychotic outcomes may arise from distinct polygenic–environmental constellations: schizophrenia-related liability appears to preferentially bias neurodevelopmental and negative symptom trajectories, whereas mood disorder–related polygenic profiles, when coupled with childhood trauma, may favor affective-reactive pathways to psychosis. Identifying these alternative pathways may enable clinicians to delineate more precisely targeted intervention points early in the course of illness or during the psychotic risk syndrome, thereby informing preventive strategies. An integrated assessment of genetic and traumatic factors may enable more precise stratification of at-risk individuals and the personalization of preventive and therapeutic interventions.

### Genomically focused models: timing, epigenetic embedding, and penetrance modulation

A central contribution of genomically focused models is to specify how polygenic liability can become developmentally expressed, and why the same liability may lead to different outcomes depending on timing and context.

*Critical Window Neurodevelopmental Model.* A complementary perspective emphasizes the timing of risk exposure and the developmental sequence of brain processes. Common and rare genetic variants may exert different effects depending on the neurobiological window involved (e.g., neuronal migration (Bradshaw and Porteous 2012), GABAergic inhibitory maturation (Benes and Berretta 2001), or synaptic pruning during adolescence (Feinberg 1982). Childhood trauma may intersect with these critical windows, inducing synaptic remodeling and connectivity changes (Teicher et al. 2016), which, when combined with an unfavorable PRS, could shift developmental trajectories toward negative/disorganized phenotypes or, alternatively, toward affective-reactive profiles.

*Mediational Epigenetic Model (G×E→epi).* Childhood trauma may act as both a moderator and mediator of genetic effects, leading to persistent epigenetic modifications (i.e., DNA methylation, histone remodeling, miRNA) in genes involved in stress response, synaptogenesis, and neuroplasticity (Thumfart et al. 2022). Within this framework, PRS may enhance epigenomic susceptibility to specific adversities, particularly those of a traumatic nature. From this perspective, the model suggests that stable, yet partially reversible epigenetic signatures could be identified in leukocytes or saliva and serve as targets for “epigenetic-sensitive” psychosocial and biological interventions (e.g., stress-reduction programs, sleep/circadian rhythm interventions, increased physical activity) (Nishitani et al. 2025).

*Rare Variant and Modulated Penetrance Model.* Some individuals carry rare high-risk variants—such as copy number variants (CNVs) including 22q11.2, 1q21.1, and 16p11.2—whose penetrance may be modulated by PRS and, likely, by environmental factors (Bergen et al. 2019). Within this framework, trauma and cumulative environmental exposures contribute to shaping both the severity and direction of phenotypic expression—toward either a “cognitive-negative” or an “affective-reactive” form of psychosis. Based on this model, targeted CNV screening in early-onset cases with pronounced neurodevelopmental features could guide rehabilitation strategies and family support pathways (Brownstein et al. 2022).

*G×E×D Model (Gene × Environment × Development).* A cross-cutting integration considers triple interactions, where the same event (e.g., bullying at a critical age such as 11–13 years) may lead to different outcomes depending on developmental timing, genetic background, and epigenetic history (Boyce et al. 2020). This model supports the use of longitudinal platforms with periodic assessments—behavioral, biological, and digital—to model individual trajectories and implement “just-in-time” interventions (Potter et al. 2023).

Across these genomically focused models, the shared theme is that genetic effects are neither uniform nor static: they vary by developmental window, may be biologically embedded through epigenetic processes, and can be modulated in penetrance and phenotypic direction by environmental context. This perspective provides a mechanistic bridge from the introduction’s emphasis on polygenic liability to the observed heterogeneity in CHR/FEP symptom dimensions.

### Pathophysiologically focused models: convergence routes from adversity to brain systems

While genomically focused models clarify *when* and *how* liability is expressed, pathophysiologically focused models articulate *through which systems* genetic vulnerability and trauma may converge.

*Immuno-Inflammation and Microglia Model.* This model integrates neuroinflammation with peripheral immunity and the stress-response. Early trauma may promote a state of low-grade inflammation, alterations in blood–brain barrier permeability, and microglial priming, leading to increased sensitization to subsequent stressors (Ferle et al. 2020). Certain polymorphisms in immunoregulatory genes (e.g., MHC or complement system genes) may modulate microglial reactivity to environmental signals, contributing to aberrant synaptic pruning and disconnection of frontotemporal networks (Misra et al. 2018). Identifying “immune-high” subtypes—characterized by elevated CRP/cytokine levels and abnormal glial patterns—may help predict a preferential response to anti-inflammatory or add-on treatments (Upthegrove et al. 2025).

*Gut–Brain–Immune Axis Model.* Early trauma and environmental factors (i.e., diet, antibiotics, stress) alter the gut microbiota, modulating immunity, tryptophan metabolism (via the kynurenine pathway), mucosal barriers, and vagal–cortical signaling (Cryan et al. 2019). Specific microbiota profiles may interact with genetic variants related to immunometabolism, predisposing individuals to phenotypes characterized by anergia/negative symptoms or, conversely, by affective lability and stress reactivity (Wu et al. 2025). From this perspective, identifying “dysbiotic” subgroups may support the use of nutritional or microbiota-targeted interventions as adjunctive treatments (Liu et al. 2020). Although both of these immunological models partially overlap with the mediational epigenetic framework—particularly in relation to environmentally driven biological embedding—it is specifically characterized by a focus on immune-inflammatory pathways, microglial dynamics, and their role in synaptic remodeling and stress sensitization.

*Dual Pathway Dopamine–Glutamate Model.* Beyond the affective trajectory, this model proposes a dual neurotransmitter pathway, with (a) a striatal dopaminergic route sensitive to stress and affective cues (e.g., hyper-salience, emotional reactivity), and (b) a cortico-hippocampal glutamatergic route more closely linked to neurodevelopmental vulnerability and cognitive deficits (Sanacora et al. 2022). The genetic profile (multiple PRSs) and trauma load may bias the system toward one pathway or the other, thereby accounting for distinct clinical subtypes with differential responses to dopaminergic antipsychotics or glutamate-modulating strategies (e.g., hippocampal-targeted interventions, pro-cognitive treatments) (Dickinson et al. 2020). In clinical practice, MRS (Glx) spectroscopy, striatal dopamine indices, and behavioral salience measures could be used to guide personalized treatments (Egerton et al. 2021). This model partially converges with the Critical Window Neurodevelopmental Model, as developmental timing may shape dopaminergic and glutamatergic maturation and bias pathway dominance. However, the distinction remains essential: the dual pathway model emphasizes neurochemical circuitry, whereas the Critical Window model focuses on the temporal dynamics of risk exposure.

*Circadian–Sleep and Allostasis Model.* Altered circadian rhythms, along with sleep deprivation and instability, appear to exacerbate salience disruptions, emotional dysregulation, and executive dysfunction (Kim et al. 2023). In this model, genetic vulnerability modulates receptor function and clock genes, while trauma may precipitate early dysregulation of the HPA axis (Matosin et al. 2018). Consequently, sleep- and rhythm-based interventions (e.g., CBT-I, circadian hygiene, light/timing protocols) could be proposed as primary or secondary prevention strategies for high-risk individuals with elevated PRS (Palagini et al. 2024).

Collectively, these models propose plausible “convergence routes” through which polygenic liability and trauma can translate into alterations of synaptic plasticity, immune tone, neurotransmission, gut-related immunometabolism, and allostatic regulation. Importantly, they also imply that some modifiable processes (e.g., inflammation, sleep/circadian instability) may function as transdiagnostic amplifiers across distinct trajectories.

### An environmentally focused model: cumulative burden and developmental context

Environmental models broaden the lens from discrete adversities to cumulative and structured exposures, emphasizing that the developmental environment is not simply an external input but a context shaping biological and behavioral pathways.

The socioecological and cumulative exposome model considers the cumulative burden of environmental factors—such as urbanicity, minority status, pollutants, deprivation, and social isolation—across the individual’s lifespan (Pries et al. 2021). The exposome interacts with genetic profiles by influencing the HPA axis, inflammation, circadian rhythms, and risk behaviors (e.g., substance use) (Matosin et al. 2018). Phenotypic manifestation are shaped not only by the presence of exposure, but also its temporal pattern (dose, duration, synergy) and relational proximity (within family, school, and community contexts) (Brown 2011; Paquin et al. 2021). The application of exposome assessment tools and cumulative risk models integrated with PRS may help identify “environmentally sensitive” individuals with potentially modifiable trajectories (Vineis et al. 2020).

This view resonates with the introduction’s framing of childhood trauma as a determinant with lifespan impact, while also positioning trauma within a broader exposure ecology that can amplify or mitigate risk trajectories.

### Subtyping and computational models: mapping heterogeneity into actionable profiles

While the previous models specify mechanisms and exposures, subtyping and computational models provide tools to formalize heterogeneity and translate it into targetable clinical profiles.

*Symptom Network Model.* Psychosis emerges from the dynamic interaction among symptoms—such as anxiety, insomnia, referential thinking, and social withdrawal—that self-sustain within a network (Borsboom 2017; Astill Wright et al. 2023). Genes and trauma do not directly cause a unitary disorder but reshape the network’s topology (i.e., node centrality, connectivity, and activation thresholds) (Isvoranu et al. 2017). The affective pathway tends to increase the weight of emotional and stress-related nodes, while neurodevelopmental vulnerability amplifies cognitive/negative nodes (Alameda et al. 2024). According to this model, high-frequency digital monitoring (e.g., ecological momentary assessment), combined with individual network analyses to target specific nodes (e.g., insomnia or rumination), could be a strategy to prevent transition to full-blown psychotic disorder (Bell et al. 2024).

*Multimodal Subtyping Model (“Biotype”).* This approach integrates genetics, epigenetics, neuroimaging, cognitive data, immune profiles, and digital signals to identify transdiagnostic biotypes (Krčmář et al. 2023). Unsupervised algorithms may reveal clusters with specific combinations of factors (e.g., high affective PRS + severe trauma + moderate inflammation; high schizophrenia PRS + cognitive deficits + signs of hyper-pruning) (Pelin et al. 2021). In clinical settings, future protocols could explore “match-to-biotype” strategies to select the most appropriate antipsychotics, potential anti-inflammatory add-ons, psychological therapies (e.g., CBT for insomnia, emotional regulation), and targeted social interventions (Comai et al. 2025).

*Computational Predictive Model (Predictive Precision).* Psychotic symptoms may be driven by altered tuning of the precision (weighting) assigned to sensory signals relative to internal priors (Sterzer et al. 2018). Genetic influences on synaptic plasticity and neuromodulation (e.g., dopamine, acetylcholine, noradrenaline) (Mäki-Marttunen et al. 2024), in combination with trauma (Kaye and Krystal 2020; Howes et al. 2020) that increases environmental uncertainty, may lead to hyper-salient perceptual hypotheses or failures in updating predictions. In this context, computational tasks (e.g., learning under volatility), pupillometry, and EEG signals (e.g., mismatch negativity) (Bismark et al. 2024; Aich et al. 2025) could help define treatable profiles suitable for metacognitive training and interventions that reduce perceived volatility (Kafadar et al. 2020).

Across these approaches, the central gain is methodological: they offer ways to operationalize heterogeneity into measurable signatures, potentially enabling prevention strategies that are tailored not only to diagnosis but to trajectory and mechanism (See Table 1).

**Table 1:** Summary of models contributing to the Neuropsychodevelopmental framework

Model	Core mechanism	Level of Analysis	Key Clinical Implications
Critical window neurodevelopmental model	Timing-dependent effects of genetic and environmental risk during sensitive developmental periods	Developmental/neurobiological	Identification of sensitive periods for preventive interventions
Mediational epigenetic model (G×E→epi)	Environmental exposures induce epigenetic modifications influencing gene expression	Molecular/epigenetic	Targeting reversible epigenetic processes (e.g., stress, lifestyle interventions)
Rare variant and modulated penetrance model	Interaction between rare high-impact variants and polygenic/environmental modifiers	Genetic/developmental	Stratification of high-risk individuals (e.g., CNV carriers)
G×E×D model (gene × environment × development)	Triple interaction shaping trajectories depending on timing and context	Integrative/developmental	Longitudinal monitoring and timing-specific interventions
Immuno-inflammation and microglia model	Chronic inflammation and microglial priming alter synaptic pruning and stress sensitivity	Immunological/neurobiological	Identification of “immune-high” subtypes; anti-inflammatory strategies
Gut–brain–immune axis model	Microbiota-mediated modulation of immunity, metabolism, and brain signaling	Systemic/neuroimmune	Microbiota-targeted interventions (diet, probiotics)
Dual pathway dopamine–glutamate model	Imbalance between dopaminergic (affective/salience) and glutamatergic (cognitive/developmental) pathways	Neurochemical/circuit-level	Personalized pharmacological strategies (dopamine vs. glutamate modulation)
Circadian–sleep and allostasis model	Disruption of circadian rhythms and stress regulation systems	Pathophysiological/behavioral	Sleep and circadian interventions (CBT-I, light therapy)
Socioecological/exposome model	Cumulative environmental burden across the lifespan	Environmental/population-level	Identification of modifiable environmental risk factors
Symptom network model	Psychopathology emerges from dynamic interactions among symptoms	Psychopathological/computational	Targeting central symptoms (e.g., insomnia, anxiety)
Multimodal subtyping model (“biotype”)	Integration of multi-domain data to identify transdiagnostic clusters	Multimodal/data-driven	Precision psychiatry and biotype-guided treatment
Computational predictive model	Altered tuning of the precision (weighting) assigned to sensory signals relative to internal priors	Computational/cognitive	Behavioral and neurophysiological markers for stratification and metacognitive training

### Cross-cutting implications

Taken together, the models described converge on three main implications relevant to both research and clinical practice. First, personalized stratification consists in combining multiple PRSs (i.e., schizophrenia, depression, bipolar), immuno-epigenetic markers, sleep/circadian profiles, and symptom network indices. Second, targeted prevention aims at intervening on transdiagnostic modulators (i.e., sleep, inflammation, stress, social isolation) that amplify multiple trajectories. Third, treatments and outcomes should shift from one-size-fits-all protocols to biotype-guided pathways, with responses monitored through digital measures and dynamic biomarkers.

Looking ahead, the field is likely to move toward hybrid models, where the affective–genomic pathway constitutes one of the main trajectories alongside neurodevelopmental, immuno-inflammatory, socioecological, and computational profiles. The challenge will be to translate these theoretical maps into actionable clinical tools capable of predicting individual courses and guiding early, multimodal, and biotype-matched interventions.

### The neuropsychodevelopmental model

The need to move beyond a purely neurobiological view of development in favor of a more multidimensional approach leads us to propose an integrative neuropsychodevelopmental model of schizophrenia. This perspective acknowledges that brain, psychological, and relational processes form and modulate each other throughout the lifespan—particularly during childhood and adolescence, periods marked by heightened plasticity and vulnerability. The brain does not develop in isolation, but within a relational and affective context that guides its functional and structural maturation (Gee 2022; Thyen et al. 2023).

Within this framework, we use the terms “neuro-” and “psycho-” in a broad and integrative sense. The “neuro-” dimension refers to the set of biological processes underlying brain development and function, including genetic liability (e.g., polygenic risk scores), epigenetic mechanisms, neurodevelopmental processes, and system-level neural alterations such as synaptic pruning, neurotransmission, and brain connectivity. The “psycho-” dimension refers to psychological and behavioral processes emerging across development, including emotion regulation, stress reactivity, attachment patterns, cognitive schemas, and subjective experience. Importantly, these dimensions are not unidirectional: psychological processes may also shape biological systems over time—for example, through stress-related modulation of the HPA axis, epigenetic changes, and neuroplastic adaptations. Accordingly, the “neuro-” and “psycho-” domains are conceptualized as dynamically interacting and mutually influencing systems that co-evolve across development and jointly contribute to the emergence of psychopathological trajectories.

In fact, psychological processes—such as emotion regulation, attachment, identity formation, mentalization, and stress resilience—emerge from continuous interactions between genetic predispositions and the quality of the developmental environment (Fine and Sung 2014; Beauchaine et al. 2018). From this viewpoint, early experiences—especially traumatic ones—are not merely environmental risk factors but active modulators of neurobiological development, capable of shaping brain connectivity, stress reactivity, and the organization of emotional–cognitive circuits (Meyer and Lee 2019; Gee 2022).

The proposed neuropsychodevelopmental model also builds upon the traditional biopsychosocial model in medicine (Engel 1977) as applied to schizophrenia, while placing a specific emphasis on the developmental organization of the person. Within this framework, the psyche is conceptualized as the interface through which neurobiological processes continuously interact with the individual’s relational, social, and cultural environment, allowing social determinants to become biologically and developmentally embedded across the lifespan.

Applied to the understanding of schizophrenia, the neuropsychodevelopmental paradigm allows us to interpret the disorder not solely as the outcome of an early lesion or a predetermined genetic deviation, but as the result of a dynamic developmental process in which brain, psyche, and environment continuously interact (Rapoport et al. 2012). Genetic vulnerability and childhood trauma act synergistically, modulating neuropsychological and affective pathways that may, over time, converge toward psychotic outcomes (Walker et al. 2013; Garcia-Rizo and Bitanihirwe 2025). The integration of biological and psychological dimensions also helps explain the wide heterogeneity of clinical phenotypes and developmental trajectories: individuals with similar genetic load may develop very different clinical profiles depending on relational experiences and the environmental support received (Howes and Murray 2014; Mennigen and Bearden 2020) Fig. 1.

**Fig. 1:**
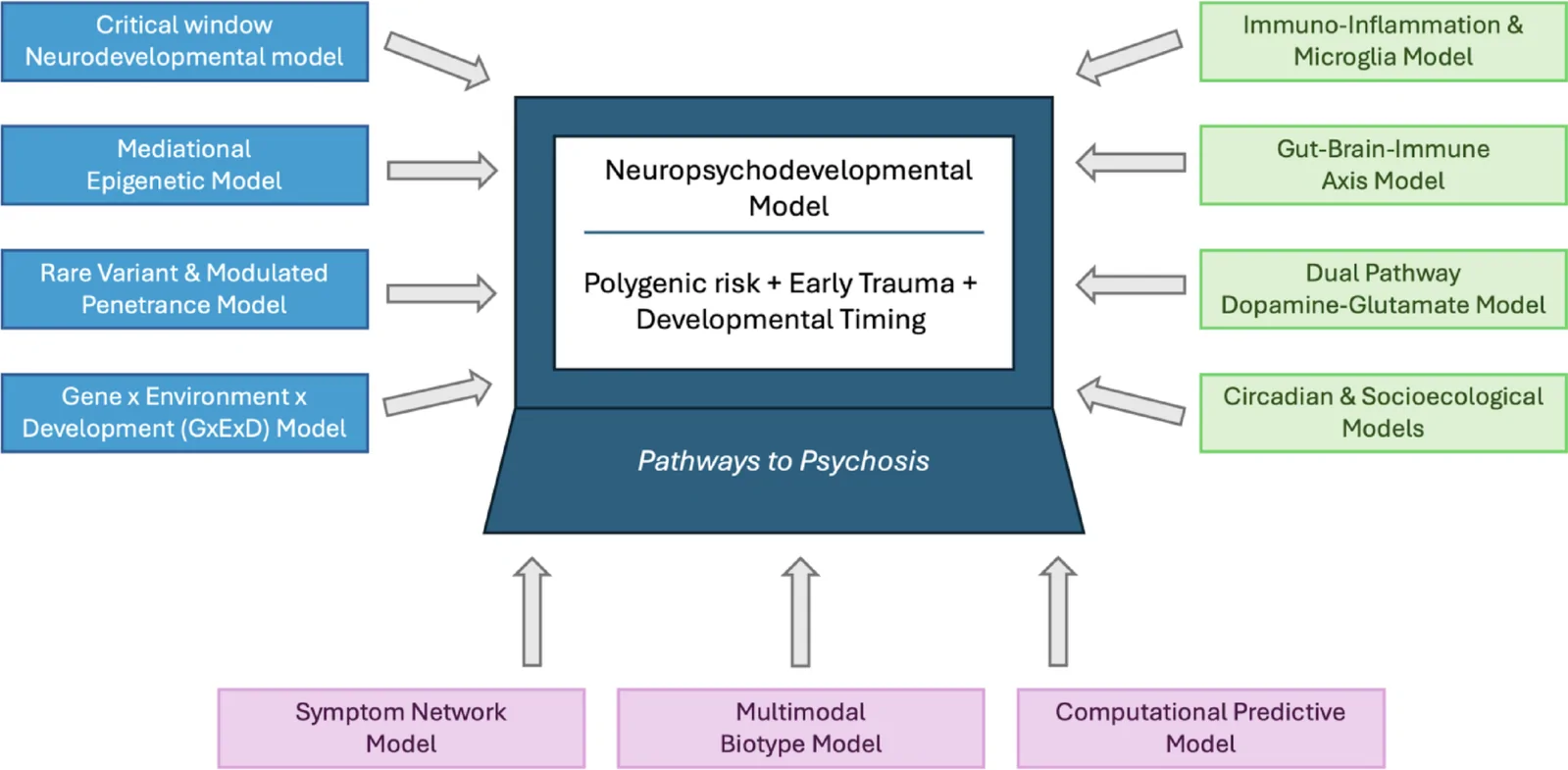
Integration of multilevel models within the Neuropsychodevelopmental framework of psychosis. This schematic illustrates how different theoretical models of schizophrenia converge within a unified Neuropsychodevelopmental Model. Genomically focused models (left; e.g., Critical Window Neurodevelopmental, Mediational Epigenetic, Rare Variant/Modulated Penetrance, and G×E×D) define how genetic liability is developmentally expressed and modulated over time. Pathophysiologically focused models (right; e.g., Immuno-Inflammation and Microglia, Gut–Brain–Immune Axis, Dual Dopamine–Glutamate Pathway, and Circadian/Allostatic models) specify the biological systems through which genetic and environmental factors are instantiated. Downstream, computational and subtyping approaches (bottom; e.g., Symptom Network, Multimodal “Biotype,” and Computational Predictive models) provide tools to operationalize heterogeneity and translate it into clinically actionable profiles. At the center, the Neuropsychodevelopmental Model integrates these dimensions, conceptualizing psychosis risk as the result of dynamic interactions among polygenic liability, developmental timing, and environmental exposures—including early trauma, broader ambient context, and psychological processes such as emotion regulation, attachment, and stress reactivity. The convergence of these multilevel processes gives rise to distinct pathways to psychosis, accounting for clinical heterogeneity and supporting stratified and personalized intervention strategies

From this perspective, schizophrenia could be considered as a neuropsychodevelopmental disorder, in which genetic, epigenetic, and environmental factors intertwine to determine not only the risk of onset, but also the form, severity, and reversibility of symptoms (Kofink et al. 2013; Zwicker et al. 2018; Smigielski et al. 2020). The concept of neuropsychodevelopment thus encourages us to view psychotic vulnerability as a dynamic and modifiable process, and to intervene early to support the harmonious development of mind and brain (Keshavan et al. 2014; Meyer and Lee 2019; Patel et al. 2021). This vision paves the way for an evolutionary and preventive psychiatry (Howes and Murray 2014; Meyer and Lee 2023), capable of integrating biological and psychological strategies to foster resilience, promote mental health, and reduce the impact of genetic vulnerability across the lifespan.

### Future directions

The integration of genetic and environmental data into psychosis risk assessment offers a tangible route toward precision psychiatry, yet several methodological and interpretative constraints currently limit translation. First, available PRSs are still largely derived from European-ancestry cohorts, restricting ethnic and cultural representativeness and potentially compromising both calibration and clinical equity. Second, polygenic scores primarily capture common variation (minor allele frequency > 1%), thereby underrepresenting rare variants and overlooking epigenetic interactions that may substantially modulate developmental risk. Third, despite their conceptual appeal, PRSs remain far from routine clinical implementation, in part because their predictive value is modest when applied in isolation.

A central future direction is therefore integration: combining PRS with environmental burden, neurocognitive vulnerability, and neurobiological indicators to increase predictive resolution and to identify those individuals most likely to benefit from targeted preventive interventions. Within this integrative approach, a particularly promising line of inquiry concerns the possibility that childhood trauma is not only an interacting exposure but also a biological “embedding” mechanism, potentially mediating long-lasting epigenetic modifications that affect gene expression and stress reactivity (Loureiro et al. 2021; Reynolds 2021). Operationally, this calls for multi-omic designs capable of linking developmental exposures to mechanistic intermediate phenotypes and, ultimately, to clinical trajectories.

Accordingly, the next frontier lies in multi-layered, longitudinal models that combine genetics, epigenetics, transcriptomics, and brain imaging within developmentally informed designs. Such approaches can move the field beyond static associations toward mechanistic and temporally structured explanations of how early experiences and polygenic liability converge to shape symptom dimensions and illness progression. Importantly, these models may enable risk stratification that is clinically interpretable—anchored in modifiable processes and matched to preventive targets.

## Conclusions

In conclusion, the PRS for schizophrenia represents a powerful tool for indexing polygenic liability and for advancing mechanistic understanding of psychotic vulnerability. However, its interpretation requires explicit consideration of environmental context, particularly childhood trauma, since genetic liability does not unfold in a vacuum. The interaction between genes and trauma does not follow a single causal direction: in some cases, trauma amplifies the phenotypic expression of genetic vulnerability; in others, it may modify or even reverse association patterns. This non-linearity reinforces the need to move away from simple additive models and toward developmentally informed accounts of risk expression.

Emerging findings also highlight the clinical relevance of affective dimensions in the pathogenesis of the psychosis, especially when mood disorder-related polygenic vulnerabilities co-occur with early trauma histories. Together, these observations support a dimensional and integrated view of psychopathology—one that moves beyond rigid diagnostic dichotomies and instead emphasizes multiple pathways converging on psychotic outcomes through distinct symptom configurations.

From this perspective, heterogeneity should not be regarded as a limitation of psychiatric classification, but as a clinically informative signal reflecting multiple developmental routes to psychosis, each associated with partially distinct genetic liabilities, environmental exposures, and modifiable mechanisms.

Looking ahead, neuropsychodevelopmental models that integrate polygenic risk, environmental exposures, psychopathology, and developmental timing offer a coherent framework for explaining heterogeneity and for identifying clinically meaningful targets. The central translational challenge will be to convert the complexity of genetic and environmental information into actionable clinical tools that can guide early identification, personalize prevention, and inform treatment selection based on individual trajectories rather than diagnosis alone.

## Data Availability

No datasets were generated or analysed during the current study.
